# Mesoscopic Structure of Lipid Nanoparticles Studied by Small-Angle X-Ray Scattering: A Spherical Core-Triple Shell Model Analysis

**DOI:** 10.3390/membranes15050153

**Published:** 2025-05-16

**Authors:** Hao Li, Panqi Song, Yiwen Li, Shuyang Tu, Mehwish Mehmood, Liang Chen, Na Li, Qiang Tian

**Affiliations:** 1 State Key Laboratory of Environment-Friendly Energy Materials, School of Materials and Chemistry, Southwest University of Science and Technology, Mianyang 621010, China; 2 National Facility for Protein Science Shanghai, Shanghai Advanced Research Institute, Chinese Academy of Sciences, Shanghai 201210, China; 3 Institute of Nuclear Physics and Chemistry, China Academy of Engineering Physics, Mianyang 621999, China

**Keywords:** lipid nanoparticles, drug delivery system, small-angle X-Ray scattering, core-triple shell model, model simulation

## Abstract

Lipid nanoparticles (LNPs) are widely recognized as effective drug delivery systems for RNA therapeutics because their efficacy is critically dependent on structural organization. The mesoscopic architecture of these multicomponent systems, which is governed by interactions among ionizable lipids, structural lipids, nucleic acids, and stabilizers, dictates encapsulation efficiency, biodistribution, and therapeutic performance. Although small-angle X-Ray scattering (SAXS) enables nanostructure characterization, the absence of suitable analytical models has hindered LNP development. Here, we present a core-triple shell SAXS model that resolves LNP hierarchical organization, including the inner lipid layer, intermediate hydrophilic region, and outer PEG corona. For LNPs encapsulating mRNA, a Gaussian distribution model was implemented to characterize the quasi-periodic structure originating from the self-assembly of mRNA-ionizable lipid complexes. Validation studies employing Comirnaty-based LNPs demonstrated that controlled variation of nitrogen-to-phosphorus (N/P) ratios produced distinguishable structural features that establish quantitative correlations between N/P ratios and LNP mesoscopic assembled structure. The modeling framework provides pharmaceutical researchers with robust analytical tools for systematic stability assessment and precision formulation for the optimization of LNPs. These structural insights are expected to advance the development of next-generation RNA therapeutics by potentially enhancing their delivery efficiency and pharmacokinetic properties.

## 1. Introduction

Lipid nanoparticles (LNPs) have revolutionized modern medicine as versatile carriers for therapeutic agents, bridging the gap between drug discovery and clinical application. Their history as drug delivery systems dates back to the early 1990s, when they emerged as alternatives to traditional carriers such as liposomes [1,2,3]. Initially designed to enhance the stability and bioavailability of hydrophobic drugs, LNPs have evolved to incorporate various lipids, particularly ionizable cationic lipids that are essential for nucleic acid delivery [1,4]. By the early 2000s, LNPs became promising carriers for gene therapy, protecting nucleic acids from degradation and enhancing cellular uptake. The successful formulation of siRNA therapies, exemplified by the FDA approval of Onpattro in 2018, highlighted the potential of LNPs in nucleic acid delivery [5,6]. The COVID-19 pandemic accelerated the clinical translation of nucleic acid-based therapeutics, as shown by the successful development of mRNA vaccines [7,8,9]. This advance highlights the potential of LNPs as a promising delivery platform for nucleic acids, as demonstrated by the regulatory approval of mRNA vaccines from Pfizer-BioNTech and Moderna [9,10,11]. These achievements have accelerated research into LNP applications for a wide range of therapeutic applications for treating cancer, genetic disorders, and other diseases [12,13,14,15,16].

The remarkable efficacy of LNPs arises from their multicomponent architecture, which enables precise control over drug delivery. These systems integrate ionizable cationic lipids, cholesterol, helper phospholipids, and polyethylene glycolated (PEGylated) lipids through sophisticated intermolecular interactions [10,17]. The functional efficacy of LNPs primarily depends on the synergistic effects of their structural components [18,19]. Ionizable cationic lipids, with their pH-dependent charge characteristics, play a pivotal role in mRNA encapsulation and endosomal escape [20,21]. Cholesterol regulates membrane fluidity and stability, while helper phospholipids (e.g., DSPC) ensure proper bilayer formation and structural organization [17,22]. PEGylated lipids, through their surface modification, significantly enhance the stability and circulation time of LNPs in biological systems [23,24,25]. These carefully engineered interactions directly influence the therapeutic efficacy of LNP-based delivery systems, underscoring the need for a deeper understanding of their mesoscopic structure.

Despite their clinical success, characterizing the complex structure of LNPs remains a significant challenge in the field. Given the intricate structure-function relationship of LNPs, comprehensive characterization of their mesoscopic structure remains crucial yet challenging. Advanced analytical techniques are required to elucidate the complex interactions among components and their impact on drug delivery efficiency. This structural understanding is essential for the rational design and optimization of LNP-based therapeutic systems, particularly in the context of mRNA delivery and targeted therapies. Dynamic light scattering (DLS) is capable of measuring particle size and polydispersity. However, it can only provide the overall hydrodynamic diameter of LNPs, which can be affected by surface charge and heterogeneity of measured systems, leading to the ambiguity of the particle size distribution [26]. Cryogenic transmission electron microscopy (cryo-TEM) is another suitable technique for observing the morphology of LNPs, but it fails to distinguish between the drug-free LNPs and mRNA-loaded LNPs. Furthermore, the cryo-TEM is constrained by a restricted observable region of interest, which results in statistical limitations [26,27]. Moreover, sample preparation for cryo-TEM may introduce biases that further complicate the data interpretation. Due to these shortcomings, there is a need for rapid, high-throughput structural characterization technique that can be taken under physiological conditions.

Small-angle X-Ray scattering (SAXS) is a powerful tool to address these challenges, offering unique insights into the nanoscale organization of LNPs [28,29,30]. This technique can probe the intra- and inter-nanoparticle structures at a scale ranging from 1 to 200 nm [31]. When combined with synchrotron radiation sources, the performance of SAXS can be enhanced with high throughput and high spatial resolution, allowing for in-depth mesoscopic structural characterization of LNPs. Synchrotron SAXS enables real-time monitoring of dynamic processes for LNPs systems, such as the assembly and disassembly of nanoparticles, nucleic acid encapsulation, and nucleic acid release mechanisms. This is particularly important for understanding how LNPs respond to environmental changes, such as pH or temperature shifts, which are relevant to their manufacturing and function in vivo. Over the past four decades, extensive studies using SAXS have revealed that liposomes typically exhibit a continuous, symmetric bilayer organization [32,33,34,35,36,37,38,39]. In contrast, LNPs possess a more complex architecture, characterized by a core-shell structure with heterogeneous and dynamic membrane layers. Moreover, the encapsulation of RNA in LNPs leads to the formation of a distinct periodic structure within the core, which simultaneously influences the organization and properties of the surrounding membrane layers [40,41,42]. Yanez et al. employed small-angle neutron scattering (SANS) to elucidate the internal architecture of LNPs, proposing a structural model consisting of a core surrounded by two distinct shells [43]. Recently, Gilbert et al. combined cryo-TEM with SAXS to analyze the structure of nucleic acid-loaded lipid nanoparticles employing a core-shell sphere model [44]. Unruh et al. explored a core and two shells model for hybrid lipid-polymer nanoparticles [45]. While these studies have significantly contributed to our understanding of LNP organization, further refinement of the current structural models is required to fully capture the complexity and dynamic nature of LNPs.

Herein, we propose a spherical core-triple shell model that characterizes the mesoscopic organization of LNPs by analyzing membrane structural parameters and periodic features. The model’s applicability to LNPs was validated with different nitrogen-to-phosphorus (N/P) ratios within the formulation guidelines of the FDA-approved Comirnaty vaccine. The structural insight into composition-dependent changes to structure provides a foundation for the rational design of nucleic acid carriers with enhanced therapeutic performance.

## 2. Materials and Methods

The LNPs were prepared in accordance with the Comirnaty formulation (Pfizer-BioNTech, New York, NY, USA) [11,46,47]. The materials utilized for the synthesis of the samples included [(4-hydroxybutyl) azanediyl] di(hexane-6,1-diyl) bis(2-hexyldecanoate) (ALC-0315, purity: ≥98%, MedChemExpress, Princeton, NJ, USA), methoxypolyethyle-neglycoloxy (2000) -N,N-ditetradecylacetamide (ALC-0159, ≥95%, Cayman Chemical, Ann Arbor, MI, USA), 1,2-distearoyl-sn-glycero-3-phosphocholine (DSPC, purity: ≥98%, Cayman Chemical), cholesterol (Ph. Eur., Sigma-Aldrich, Darmstadt, Germany), and ethanol (absolute for analysis, p.a., Sigma-Aldrich, Darmstadt, Germany). mRNA was transcribed in vitro from a 3800 nt PCR-amplified dsDNA template using T7 RNA polymerase (M0251L, New England Biolabs, Ipswich, MA, USA) and subsequently purified using Monarch RNA cleanup kit spin columns (T2040L, New England Biolabs, Ipswich, MA, USA).

The chemical composition of the prepared LNPs was based on the following molar ratio: ALC-0315:DSPC:cholesterol:ALC-0159 = 48.2:10:40:1.8. The molar ratio of the amine groups on the ionizable lipid to the phosphate groups on mRNA (N/P ratio) was adjusted to 3:1, 5:1, 6:1, 7:1, and 8:1, respectively. When the N/P ratio was altered, the concentration of mRNA was kept constant, while the concentrations of DSPC, cholesterol, and ALC-0159 were adjusted in proportion to the concentration of ALC-0315. Ethanolic lipid mixtures were prepared by dissolving ALC-0315, ALC-0159, DSPC, and cholesterol in ethanol. Two types of buffers were prepared for the formulation of LNPs. The first buffer, pH 7.4, was prepared by dissolving the following components in ultrapure water: sodium chloride (8.01 g/L), potassium chloride (0.20 g/L), dibasic sodium phosphate dehydrates (1.42 g/L), and monobasic potassium phosphate (0.24 g/L). The second buffer, pH 4.0, was prepared by dissolving citric acid (13.10 g/L) and sodium citrate (6.90 g/L) in ultrapure water (Millipore, Bedford, MA, USA). For the preparation of mRNA-loaded LNPs (mRNA-LNPs), the mRNA was dissolved in the aqueous buffer at pH 4.0. This facilitated the encapsulation of mRNA within the LNP structure. For drug-free LNPs (empty-LNPs), the aqueous phase was formulated without the addition of mRNA. Both types of LNPs were produced using a microfluidic mixer (MicroNano, Suzhou, China) under ambient conditions. The mixing was conducted at a flow rate of 12 mL/min, with a volume ratio of 3:1 (organic phase to aqueous phase). After the initial formation, the resulting LNPs underwent an overnight buffer exchange against PBS buffer (pH 7.4). This was achieved with a Slide-A-Lyzer Dialysis Cassette (MWCO, 50 kDa) at 4 °C. Finally, the LNPs were stored at 4 °C for characterization.

The SAXS measurements were recorded at BL19U2 beamline of the National Facility for Protein Science Shanghai (NFPS), located within the Shanghai Synchrotron Radiation Facility (SSRF). The incident X-Ray beam was operated at 12 keV, corresponding to a wavelength (λ) of 0.103 nm. Two-dimensional scattering patterns were collected using a PILATUS3X 2M detector (Dectris, Baden-Daettwil, Switzerland). The sample-to-detector distance was set at 2675 mm, enabling detection of the scattering vector *q*, defined as *q* = 4πsin*θ*/*λ*, where *θ* represents half of the scattering angle, over a range of 0.07–4.5 nm^−1^. A flow cell made of a quartz capillary with an inner diameter of 1.5 mm was used to sample an aliquot of the LNP solution. The exposure time was fixed at 1 s per scattering pattern, and 20 consecutive scattering patterns were collected and compared for data averaging. Preliminary data reduction and processing were performed using the BioXTAS RAW software (version 2.2.1) package [48]. The data were fitted to analytical model functions using the method of least squares and the SASfit software (version 0.94.12) [49].

The morphology of LNPs was examined using cryo-TEM. All images were collected on a Tecnai G2 F20 200 kV TEM (FEI, Hillsboro, OR, USA) equipped with a Ceta 16M CMOS camera (FEI, Hillsboro, OR, USA). Vitreous specimens were prepared on copper grids (Quantifoil R1.2/1.3, 200 mesh Cu holey carbon, Jena, TH, Germany). Prior to sample preparation, the grids were glow-discharged for 30 s with a mixture of H_2_ and O_2_ using a plasma cleaning system (Solarus II Plasma Cleaner, Gatan, Inc., Pleasanton, CA, USA). Typically, 3 μL of the solution was applied onto the grid under controlled environmental conditions (8 °C and 100% relative humidity) and blotted with a filter paper to form a thin liquid film. The automatically blotted sample was immediately plunged into liquid ethane using a Vitrobot Mark IV (FEI, Hillsboro, OR, USA). The sample temperature was maintained at −170 °C during data collection, and observations were made in low-dose mode to minimize radiation damage from the electron beam.

## 3. Results

### 3.1. Model and Simulation

**Figure 1:**
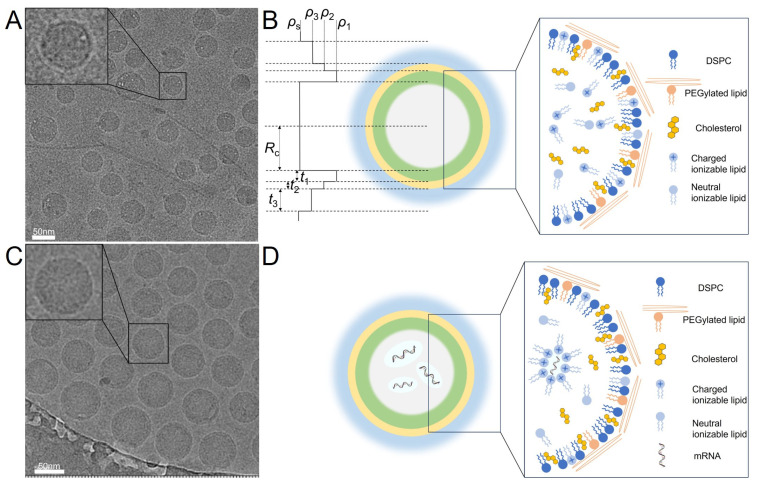
The mesoscopic structure of LNP: (**A**) Cryo-TEM micrograph of empty LNP; (**B**) Schematic representation of empty LNP; (**C**) Cryo-TEM micrograph of mRNA-LNP; (**D**) Schematic representation of mRNA-LNP.

The cryo-TEM images show that the LNPs are spherical entities, with sizes ranging from 20 to 60 nm (Figure 1A,C). No significant difference was observed between the images of mRNA-LNPs (Figure 1C) and empty-LNPs (Figure 1A). Upon further examination, a pale white shell and a darker shell surrounding the LNPs were observed, suggesting a core-shell vesicular architecture. However, due to the slender shell structure and a low electron density contrast, detailed structural information via cryo-TEM was limited. To address this limitation, Arteta et al. applied contrast variation SANS to analyze the internal structure and lipid distribution within LNPs [43]. Their findings demonstrated that LNPs are organized into a core surrounded by two concentric shells: the inner shell comprises DSPC, a fraction of DLin-MC3-DMA, cholesterol, and the DMPE moiety of PEG lipids, while the outer shell is predominantly composed of PEGylated lipids. SANS data obtained from LNPs with varied deuteration revealed that ionizable lipid MC3 and nucleic acids are predominantly localized in the core region, while DSPC and cholesterol are primarily distributed in the surrounding shell [50]. However, due to the relatively low neutron flux, coupled with the high incoherent scattering background, the construction of sophisticated models based on SANS data presents considerable difficulties.

Herein, we proposed a polydisperse spherical core-triple shell model based on the LNP system composed of ALC-0315, cholesterol, DSPC, and ALC-0159. ALC-0315, the most abundant component, is an ionizable lipid featuring a pH-responsive amine group and amphiphilic structure. The lipid forms the core and inner shell of LNPs, dynamically interacting with other components to create a well-organized nanoparticle matrix. Cholesterol, the second most abundant component, is predominantly hydrophobic with a rigid steroid structure. It stabilizes the inner lipid layer by interacting with ALC-0315 and helper phospholipid DSPC, while preventing excessive packing of phospholipid molecules to maintain optimal membrane fluidity. The DSPC, due to its saturated chains, contributes to a rigid and less permeable layer, where the hydrophilic headgroups of DSPC and ALC-0315 align to form a stable interface. The ALC-0159, with its hydrophilic PEG chains, extends outward to form a hydrated outer shell, enhancing LNP stability and preventing inner layer integration. This triple-shell structure—comprising an inner lipid layer, an intermediate hydrophilic headgroup layer, and an outer PEG shell—provides an effective framework for understanding LNP organization and function.

The scattering amplitude of a core-triple shell nanoparticle, as derived from small-angle scattering theory, is the Fourier transform of its scattering length density (SLD) [50,51,52,53]. Based on a nanoparticle dispersed in a homogeneous liquid medium with SLD of *ρ*_s_, the scattering amplitude is written as

(1) $$\begin{array}{ll} A\left(q\right) & =\int \rho (r)e^{-iq\cdot r}dr\\ & =\int_{0}^{R_{c}}\rho_{c}e^{-iq\cdot r}dr+\int_{R_{c}}^{R_{c}+t_{1}}\rho_{1}e^{-iq\cdot r}dr+\int_{R_{c}+t_{1}}^{R_{c}+t_{1}+t_{2}}\rho_{2}e^{-iq\cdot r}dr+\int_{R_{c}+t_{1}+t_{2}}^{R_{c}+t_{1}+t_{2}+t_{3}}\rho_{3}e^{-iq\cdot r}dr-\int_{0}^{R_{c}+t_{1}+t_{2}+t_{3}}\rho_{s}e^{-iq\cdot r}dr+\rho_{s}\delta (q)\\ & =\int_{0}^{R_{c}}(\rho_{c}-\rho_{1})e^{-iq\cdot r}dr+\int_{0}^{R_{c}+t_{1}}(\rho_{1}-\rho_{2})e^{-iq\cdot r}dr+\int_{0}^{R_{c}+t_{1}+t_{2}}(\rho_{2}-\rho_{3})e^{-iq\cdot r}dr+\int_{0}^{R_{c}+t_{1}+t_{2}+t_{3}}(\rho_{3}-\rho_{s})e^{-iq\cdot r}dr+\rho_{s}\delta (q) \end{array}$$

where *R*_c_ is the core radius; *t*_1_, *t*_2_, and *t*_3_ are the shell thicknesses of the inner, intermediate, and outer shell, respectively; and *ρ*_1_, *ρ*_2_, and *ρ*_3_ represent the SLD of each shell. Figure 1B shows a schematic representation of the LNP structure, accompanied by an enlarged view to highlight the intricate internal organization of the LNP. Since it is an isotropic scattering system, the first integral item of the above function can be expressed as

(2) $$\begin{array}{ll} \int_{0}^{R_{c}}(\rho_{c}-\rho_{1})e^{-iq\cdot r}dr & =(\rho_{c}-\rho_{1})\int_{0}^{R_{c}}4\pi \frac{\sin qr}{qr}r^{2}dr\\ & =(\rho_{c}-\rho_{1})\frac{4\pi {R_{c}}^{3}}{3}\frac{3[\sin qR_{c}-qR_{c}\cos(qR_{c})]}{{\left(qR_{c}\right)}^{3}}\\ & =(\rho_{c}-\rho_{1})V(R_{c})j(qR) \end{array}$$

Accordingly, the scattering amplitude of a core-triple shell nanoparticle is deduced as (*q* ≠ 0)

(3) $$\begin{array}{ll} A\left(q\right)= & \left(\rho_{c}-\rho_{1})V(R_{c})j(q,R_{c})+(\rho_{1}-\rho_{2})V(R_{c}+t_{1})j(q,R_{c}+t_{1}\right)\\ & +(\rho_{2}-\rho_{3})V(R_{c}+t_{1}+t_{2})j(q,R_{c}+t_{1}+t_{2})\\ & +(\rho_{3}-\rho_{s})V(R_{c}+t_{1}+t_{2}+t_{3})j(q,R_{c}+t_{1}+t_{2}+t_{3}) \end{array}$$

This model delineates layers from the core to the outer shells, which represent the inner lipid layer, the hydrophilic headgroup layer, and the hydrated PEG layer. For a collection of dilute core-triple shell nanoparticles dispersed in a solvent with a normal distribution *N*(*R*_c_), the scattering intensity at small angles is written as

(4) $$I(q)=\int_{0}^{\infty }N(R_{c}){\left[A(q)\right]}^{2}dR+Bg$$

(5) $$N(R_{c})=\frac{n}{\sigma \sqrt{2\pi }}\exp\left(-\frac{{\left(R_{c}-R_{0}\right)}^{2}}{2\sigma^{2}}\right)$$

where *R*_0_ is the mean radius of the core; *σ* is the standard deviation; *n* is the total number of the particles per unit volume, and *Bg* is the background.

**Figure 2:**
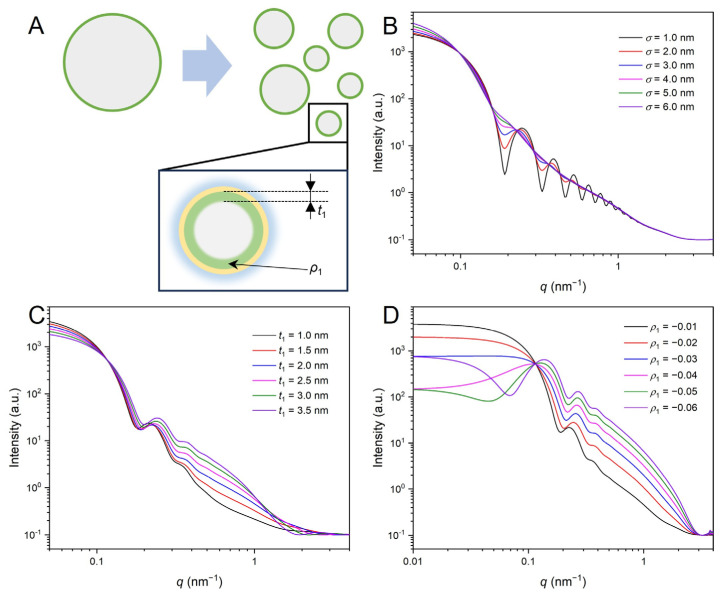
Simulation results of the core-triple shell model where *R*_0_ = 25 nm, *t*_2_ = 0.3 nm, *t*_3_ = 4 nm, *ρ*_c_ = 0.01, *ρ*_2_ = 0.01, *ρ*_3_ = 0.001, and *ρ*_s_ = 0 (the unit of SLD is arbitrary units): (**A**) Schematic representation of the polydisperse LNP; (**B**) Effect of σ on the scattering curves with *t*_1_ = 2 nm, and *ρ*_1_ = −0.01; (**C**) Effect of *t*_1_ on the scattering curves with *ρ*_1_ = −0.01; and (**D**) Effect of *ρ*_1_ on the scattering curves with *t*_1_ = 2 nm.

The model simulation results are presented in Figure 2. Since that polydispersity is a typical feature of LNPs (Figure 1A,C), the standard deviation *σ*, which accounts for the variation in core size (*R*_0_ = 25 nm), was examined. As shown in Figure 2B, with the increase in σ from 1.0 nm to 6.0 nm, the oscillation peaks gradually diminish, eventually becoming indistinguishable when *σ*/*R*_c_ exceeds 0.2. The inner shell plays a crucial role in determining the overall stability and drug-encapsulation efficiency of LNPs. Therefore, it is essential to understand the effects of the thickness (*t*_1_) and scattering length density (*ρ*_1_) for the inner multicomponent lipid layer on the scattering profiles. As shown in Figure 2C, an increase in *t*_1_ results in a shift of the first oscillation peak towards the high *q* region, and an increase in intensity at the *q* range of 0.2 to 1 nm^−1^, while simultaneously leading to a decrease in forward scattering intensity at *q* < 0.1 nm^−1^. Since the inner shell comprises a complex mixture of lipids and cholesterol (Figure 1D), the *ρ*_1_ reflects a statistical average of both hydrophobic hydrocarbon chains and other components. Therefore, *ρ*_1_, relative to the *ρ*_s_ of solvent, was assigned negative values ranging from −0.01 to −0.06, consistent with the dominant contribution from the hydrophobic regions of the lipid mixture [32,39]. The results shown in Figure 2D indicate that the scattering profiles exhibit significant sensitivity to changes in *ρ*_1_, particularly at *q* < 0.2 nm^−1^. Notably, when *ρ*_1_ is less than −0.04, a broad peak appears at about 0.1 nm^−1^.

We also simulated the effects of *t*_2_ and *ρ*_2_ (thickness and scattering length density for the intermediate hydrophilic headgroup layer), as well as *t*_3_ and *ρ*_3_ (thickness and scattering length density for the outer PEG corona), on the scattering profiles of LNPs (Figure S1). Since *t*_2_ corresponds to the thickness of the hydrophilic headgroup layer of the lipids and *ρ*_3_ represents the SLD of the hydrated PEG layer, their impacts on the scattering profile are not as significant as those of *t*_1_ and *ρ*_1_. These simulation results are crucial for applying the least-squares method to fit the experimental scattering profiles, particularly in setting the initial values for the fitting parameters.

### 3.2. Empty-LNPs and mRNA-LNPs

**Figure 3:**
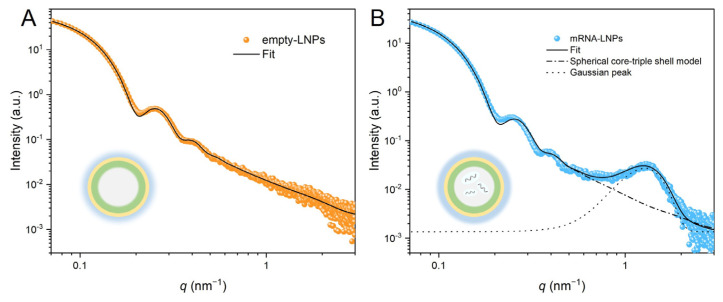
Experimental and fitted SAXS curves of LNPs: (**A**) empty-LNPs, (**B**) mRNA-LNPs. The insets show schematic representations of empty-LNPs and mRNA-LNPs.

The scattering data obtained from empty-LNPs are shown in Figure 3A. Two distinct oscillation peaks, attributed to the form factor of LNPs, are observed in the scattering curve, indicating that the LNPs exhibit good monodispersity. Due to the various parameters in Equation (4), essential approximations were made based on the prior information: (1) *ρ*_s_ was set to 0; (2) the thickness (*t*_3_) of the hydrated PEG-2000 layer was fixed at 4 nm; and (3) the thickness (*t*_2_) of the hydrophilic headgroup layer was fixed at 0.3 nm. The core-triple shell model, fitted to the scattering data of the empty-LNPs, revealed an average core radius (*R*_0_) of 22.94 nm with a standard deviation (*σ*) of 2.50 nm, and the statistically averaged thickness (*t*_1_) of the inner shell was 2.04 nm (Table 1).

The scattering data obtained from mRNA-LNPs are depicted in Figure 3B. The oscillation peaks observed in the low *q* region are consistent with those of empty-LNPs, while a distinct broad peak appears at *q* = 1.2 nm^−1^, which is attributed to the assembled structure of nucleic acids and ionizable lipids [26,43,54,55]. To describe this quasi-periodic structure, a Gaussian function (Equation (S1)) was introduced, providing a mathematical model to capture the structure of the assemblies. The SAXS data were fitted using the spherical core-triple shell model combined with a Gaussian function. The mRNA-LNPs had an average core radius of 22.01 nm with a standard deviation of 2.65 nm (Table 1). The derived *σ*/*R*_0_ of mRNA-LNPs was 0.12, which was larger than that (0.10) of empty-LNPs, indicating the increase in polydispersity upon the mRNA incorporation. The scattering contrast of the intermediate shell (*ρ*_2_) of mRNA-LNPs was approximately 21% lower than that of empty-LNPs. It means that the incorporation of mRNA modified the intermolecular interactions and arrangement within the shell layers, resulting in the structural heterogeneity and decrease in *ρ*_2_. On the other hand, the scattering contrast of the outmost PEG layer (*ρ*_3_) of mRNA-LNPs was significantly higher, by a factor of 2.6, compared to the empty-LNPs. This is attributed to the shrinkage of the core after mRNA encapsulation, leading to an increase in the density of PEG chains on the surface of LNPs. The quasi-periodic structure formed by nucleic acids and ionizable lipids was well-fitted by the Gaussian function (Figure 3B). The fitted peak position (*C*_Gaussian_) was 1.27 nm^−1^, with a width (*W*_Peak_) of 0.28 nm^−1^. The calculated periodic distance, estimated by 2π/*C*_Gaussian_, was 4.94 nm. It is consistent with the previously reported value [44].

### 3.3. The Influence of N/P Ratio

**Figure 4:**
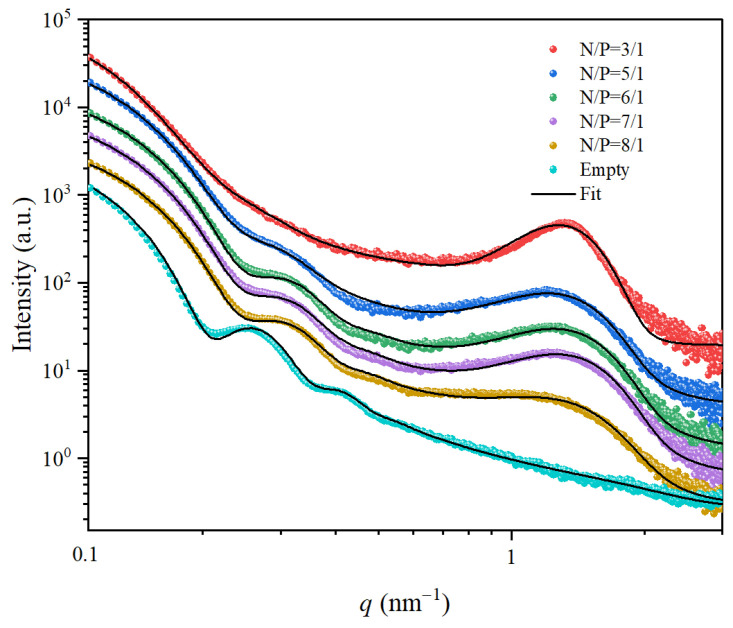
SAXS data obtained from the mRNA-LNPs with different N/P ratios. The curves are vertically shifted for clarity. The black lines are the best fits to the spherical core-triple shell model combined with a Gaussian function.

The N/P ratio, defined as the molar ratio of ionizable lipid cationic amines (N) to nucleic acid phosphates (P), serves as a critical determinant of LNP physicochemical properties including stability, encapsulation efficiency, and cellular uptake. The scattering profiles for LNPs with different N/P ratios are depicted in Figure 4. As the N/P ratio increased from 3:1 to 8:1, the oscillation peaks associated with the form factor became more pronounced. The SAXS data for different N/P ratios were fitted using the aforementioned model. The fitted parameters are presented in Table 2. At a N/P ratio of 3:1, *R*_0_ was 17.74 nm with a *σ* of 5.18 nm. As the N/P ratio increased to 8:1, *R*_0_ increased to 18.53 nm, and *σ* decreased to 3.05 nm. Notably, empty-LNPs exhibited a larger *R*_0_ of 22.13 nm with *σ* of 2.85 nm. These findings demonstrate that higher N/P ratios promote the formation of mRNA-LNPs with greater size uniformity. This phenomenon can be attributed to the competitive partitioning of ionizable lipids (ALC-0315) between membrane formation and nucleic acid binding. At lower N/P ratios, substantial amounts of cationic lipids are sequestered through electrostatic interactions with nucleic acids, thereby reducing the fraction available for proper membrane assembly and compromising structural homogeneity.

Concurrently, as the N/P ratio increased from 3:1 to 8:1, the scattering profiles exhibited two distinct trends at high *q* values: (1) a gradual decrease in intensity of the diffraction peaks between 1–2 nm^−1^, and (2) a significant broadening of peak widths from 0.26 nm⁻^1^ to 0.38 nm^−1^ (Figure 4 and Table 2). The sharper peaks (0.26 nm^−1^) at lower N/P ratios indicated more ordered periodic structures and well-organized nucleic acid-lipid complexes with uniform spacing. Conversely, the broader peaks (0.38 nm^−1^) at higher N/P ratios suggested increased structural disorder, characterized by heterogeneous packing arrangements and reduced long-range organization. This structural evolution most likely occurred because lower N/P ratios promoted electrostatic-driven ordering of lipids around nucleic acids, while higher ratios introduced excess lipids that disrupted this templated organization. The results indicate that an intermediate N/P ratio achieved an optimal balance between nucleic acid encapsulation efficiency and uniform LNP size distribution.

## 4. Discussion

The structural characterization of LNPs is critical for optimum design and performance as nucleic acid delivery systems. In this study, we employed synchrotron SAXS with a core-triple shell model to elucidate the mesoscopic structure of LNPs, to provide new insights into their assembly and nucleic acid encapsulation mechanisms. Our findings advance the understanding of LNP architecture and demonstrate how structural parameters correlate with synthesis recipes, offering a predictive understanding for rational LNP design.

Current research on LNPs has entered an era of combinatorial optimization, where lipid composition design is systematically explored. Recent studies have developed extensive libraries of ionizable lipids and optimized formulations for specific targeting applications. For instance, Su et al. designed degradable-core ionizable lipids that address stability challenges in systemic delivery [56]. Chen et al. developed a series of LNPs by modulating the structures of ionizable lipids, including the head group, tail, and linker to achieve lymph node targeting [57]. Liu et al. developed a series of novel ionizable lipids with a modular structure, where alkyl chain length modulation enabled organ-selective delivery [58]. These studies show structural control at the molecular level translates to functional performance at the nanoparticle level. However, the understanding remains incomplete because effective characterization techniques for these complex nanostructures remain limited. Cryo-TEM, while useful for visualizing LNP morphology, struggles to resolve fine structural details constrained by low electron density contrast. Similarly, SANS provides valuable insights but faces challenges in model construction due to neutron flux limitations. Our work bridges this critical methodological gap by establishing synchrotron SAXS as a powerful tool for probing LNP mesoscopic structure, enabling quantitative analysis of core dimensions, membrane thickness profiles, and SLD distributions to nanometer resolution.

The proposed polydisperse core-triple model combined with a Gaussian function, illustrated here with empty-LNPs and mRNA-LNPs, reveals several key structural features. First, the derived parameter *σ*/*R*_0_ provides a robust metric for assessing LNP uniformity. Unlike cryo-TEM, which is limited in statistical sampling, this SAXS-based method offers a robust and rapid analysis with superior statistical reliability, making it suitable for evaluating sample consistency across different preparation batches. Second, the hydrophilicity and biocompatibility of the LNPs can be assessed by the parameter *ρ*_3_, which is proportional to the density of PEG corona on the surface of LNPs. The incorporation of *ρ*_3_ is central towards understanding how the surface properties influence interactions with biological systems, ultimately affecting the delivery efficiency and therapeutic outcomes. Third, the parameters of *t*_1_, and *ρ*_1_, along with *ρ*_2_, which account for the inner and intermediate layer structure, provide insights into the stability and integrity of the LNPs. Notably, mRNA incorporation reduced *ρ*_2_ by 21%, highlighting how payload integration alters lipid organization. Fourth, the diffraction peaks in the high *q* range (1–2 nm⁻^1^) reflect the structural order of nucleic acid-lipid assemblies. That is, sharper peaks at lower N/P ratios indicate well-organized complexes, while peak broadening at higher ratios suggests disrupted organization from excess lipids. These results demonstrate that an optimal N/P ratio exists that simultaneously ensures mRNA encapsulation while maintaining structural uniformity of LNPs, enabling guidance for formulation development. By systematically varying lipid compositions and employing SAXS for structural characterization, existing LNP formulations can be optimized and novel LNPs for specific therapeutic applications developed. The correlation between SAXS-derived structural insights and key performance metrics, such as encapsulation efficiency, release kinetics, and cellular uptake, will advance our understanding of LNP design principles. Although the current model is based on the FDA-approved Comirnaty vaccine formulation, its underlying principles may extend to similar LNP systems.

This study also highlights fundamental differences between LNPs and liposomes. Liposomes are spherical nanostructures formed by self-assembly of phospholipid bilayers, typically analyzed using standard bilayer and modified bilayer models [59,60,61], where a broad peak is commonly observed at high *q*. In contrast, the membrane structure of LNPs is much more irregular, because of the presence of a high fraction of ionizable lipid and cholesterol in their formulations [21,44,54,62]. This compositional complexity creates dynamic structural behavior. First, the ionizable lipids can exist in different charge states depending on the pH of the environment. Cationic ionizable lipids not only aid the encapsulation of nucleic acids but also influence the overall arrangement of lipids within the nanoparticle. Second, neutral ionizable lipids can transition between charged and uncharged states, further complicating the lipid arrangement. Third, the inclusion of amphiphilic cholesterol into LNP formulations enhances membrane fluidity and integrity, while also contributing to the formation of complex and heterogeneous membrane architecture. In short, the irregular arrangement of lipids and cholesterol in LNPs creates fundamentally distinct scattering profiles from traditional liposomes, explaining why conventional bilayer models fail to describe LNP structures and highlighting the necessity for specialized structural models.

While this study advances LNP characterization, certain limitations must be acknowledged. The model assumes isotropic scattering and uniform SLD per shell, potentially oversimplifying lipid layer heterogeneities. Furthermore, SAXS provides only ensemble-averaged data, masking subpopulation contributions. To address these limitations, cross-validation of SAXS fitting results with cryo-TEM is strongly recommended. Another consideration in synchrotron SAXS experiments is the potential radiation damages to LNPs. In this study, we mitigated this risk by employing short exposure times (1 s per frame) and acquiring multiple frames (20 frames) for averaging. The consistency of the scattering patterns was confirmed through similarity matrix analysis of all 20 frames (Figure S2), demonstrating that radiation-induced structural alterations were negligible under our experimental conditions. This validation ensured the reliability of our SAXS-derived structural parameters. Additionally, if the LNP preparation process or storage conditions are altered (e.g., in lyophilized formulations), the scattering profiles may deviate significantly from the theoretical predictions of core-triple shell model. In such cases, alternative structural models should be considered to accurately interpret the experimental data.

In summary, this study established synchrotron SAXS as a robust method for LNP structural analysis and introduces a core-triple shell model that captures key features of mRNA encapsulation and lipid organization. By correlating structural parameters with formulation properties, we provide a roadmap for optimizing LNP design. Looking forward, integrating artificial intelligence (AI) with SAXS and other structural characterization techniques offers exciting opportunities for advancing nucleic acid drug discovery. Developing a small-angle scattering database for LNPs, along with AI-driven data analysis methods, is expected to enhance the accuracy and efficiency of SAXS model fitting, enabling more precise morphological assessments and better-quality control of LNPs. Additionally, AI can simulate and predict the effects of various LNP formulations on their structure-function relationships, enhancing the optimization process. This combination of AI and SAXS can accelerate the discovery of next-generation nucleic acid delivery systems with improved stability, targeting efficiency, and reduced toxicity.

## 5. Conclusions

This study establishes a synchrotron SAXS/core-triple shell model as a powerful approach for characterizing the mesoscopic structure of LNPs prepared by microfluidic method. Compared with the empty-LNP, the incorporation of mRNA into the core of LNPs induced the increase in polydispersity and structural heterogeneity, reflected by the increase of *σ*/*R*_0_ and the decrease of *ρ*_1_ and *ρ*_2_. The scattering contrast of the outmost PEG layer (*ρ*_3_) of mRNA-LNPs was 2.6 times higher than that of the empty-LNPs, which could be attributed to the shrinkage of the core after mRNA encapsulation. As the increase of N/P ratios, specifically from 3:1 to 8:1, mRNA-LNPs exhibited increased uniformity in particle size, although the structural periodicity of the assembled nucleic acids and lipids deteriorated, suggesting an optimal N/P ratio for efficient encapsulation. The proposed methodology helps mitigate some limitations of conventional approaches, suggesting potential applicability across various vaccine platforms. While the model simplifies certain structural complexities, it establishes a foundation for developing next-generation nucleic acid delivery systems. The future integration of AI and high-throughput synchrotron SAXS methodologies could potentially accelerate structural optimization and clinical translation of LNPs, possibly improving targeting precision while reducing toxicity.

## Figures and Tables

**Table 1:** Structural parameters of empty-LNPs and mRNA-LNPs by curve fitting using the core-triple shell model combined with a Gaussian function. *ρ*_1_, *ρ*_2_, and *ρ*_3_ are dimensionless.

Sample	*σ* (nm)	*R*_0_ (nm)	*t*_1_ (nm)	*ρ*_1_ (×10^−4^)	*ρ*_2_ (×10^−4^)	*ρ*_3_ (×10^−5^)	*C*_Gaussian_ (nm^−1^)	*W*_Peak_ (nm^−1^)
empty-LNPs	2.50 ± 0.01	22.94 ± 0.01	2.04 ± 0.02	−1.16 ± 0.02	2.77 ± 0.05	0.22 ± 0.01	-	-
mRNA-LNPs	2.65 ± 0.01	22.01 ± 0.01	1.85 ± 0.04	−1.14 ± 0.01	2.19 ± 0.05	0.55 ± 0.03	1.27 ± 0.01	0.28 ± 0.01

**Table 2:** Structural parameters of LNPs with varied N/P ratios by SAXS data fitting using the core-triple shell model combined with a Gaussian function. *ρ*_1_, *ρ*_2_, and *ρ*_3_ are dimensionless.

Sample	*σ* (nm)	*R*_0_ (nm)	*t*_1_ (nm)	*ρ*_1_ (×10^−4^)	*ρ*_2_ (×10^−4^)	*ρ*_3_ (×10^−5^)	*C*_Gaussian_ (nm^−1^)	*W*_Peak_ (nm^−1^)
3/1	5.18 ± 0.01	17.74 ± 0.03	2.57 ± 0.02	−1.07 ± 0.02	2.87 ± 0.01	1.67 ± 0.01	1.28 ± 0.01	0.26 ± 0.01
5/1	3.70 ± 0.01	17.92 ± 0.01	1.82 ± 0.02	−1.08 ± 0.01	2.96 ± 0.05	0.80 ± 0.01	1.24 ± 0.01	0.32 ± 0.01
6/1	3.12 ± 0.01	17.97 ± 0.01	1.92 ± 0.01	−1.13 ± 0.01	2.89 ± 0.04	0.63 ± 0.01	1.27 ± 0.01	0.33 ± 0.01
7/1	3.14 ± 0.01	18.23 ± 0.01	1.93 ± 0.02	−1.15 ± 0.01	2.86 ± 0.03	0.59 ± 0.01	1.29 ± 0.01	0.34 ± 0.01
8/1	3.05 ± 0.01	18.53 ± 0.01	1.87 ± 0.01	−1.18 ± 0.01	2.23 ± 0.03	0.58 ± 0.01	1.21 ± 0.01	0.38 ± 0.01
empty	2.85 ± 0.01	22.13 ± 0.01	1.91 ± 0.01	−1.23 ± 0.01	3.12 ± 0.02	0.18 ± 0.01	-	-

## Data Availability

The data presented in this study are openly available in Science Data Bank at https://doi.org/10.57760/sciencedb.23784.

## Supplementary Materials

The following supporting information can be downloaded at: https://www.mdpi.com/article/10.3390/membranes15050153/s1. Equation (S1): The expression for the Gaussian function. Figure S1: Simulation results of the core-triple shell model where *R*_0_ = 25 nm, *σ* = 3 nm, *ρ*_c_ = 0.01 and *ρ*_s_ = 0 are fixed (the unit of SLD is arbitrary units): (A) Effect of *t*_2_ on the scattering curves with *ρ*_2_ = 0.01; (B) Effect of *ρ*_2_ on the scattering curves with *t*_2_ = 0.3 nm; (C) Effect of *t*_3_ on the scattering curves with *ρ*_3_ = 0.001; (D) Effect of *ρ*_3_ on the scattering curves with *t*_3_ = 4 nm. Figure S2: SAXS profile similarity matrix for the evaluated LNPs.
