# Supplementary material for: Mesoscopic Structure of Lipid Nanoparticles Studied by Small-Angle X-Ray Scattering: A Spherical Core-Triple Shell Model Analysis
Source: Membranes (Basel). 2025 May 16;15(5):153. doi: 10.3390/membranes15050153 (PMC12113147; doi:10.3390/membranes15050153)
Supplement: Supplementary file 1 [file membranes-15-00153-s001.zip › membranes-3625825-supplementary.pdf]

Supplementary materials

Mesoscopic Structure of Lipid Nanoparticles Studied by Small-Angle X-Ray Scattering: A Spherical Core-Triple Shell Model Analysis

Hao Li [1], Panqi Song [2], Yiwen Li [2], Shuyang Tu [2], Mehwish Mehmood [1], Liang Chen [3], Na Li [2,*] and Qiang Tian [1,*]

[1] State Key Laboratory of Environment-Friendly Energy Materials, School of Materials and Chemistry,
Southwest University of Science and Technology, Mianyang 621010, China;
[2] National Facility for Protein Science Shanghai, Shanghai Advanced Research Institute, Chinese Academy of Sciences, Shanghai 201210, China
[3] Institute of Nuclear Physics and Chemistry, China Academy of Engineering Physics, Mianyang 621999, China
* Correspondence: lina02@sari.ac.cn (N.L.); tianqiang@swust.edu.cn (Q.T.)

The expression for the Gaussian function is given by:

$$f(x) = a\exp\left[-\frac{(x-x_c)^2}{2\sigma^2}\right] + c_0 \qquad \text{(S1)}$$

where, a denotes the peak amplitude of the characteristic peak, x_c indicates the position of the characteristic peak, σ represents the width of the characteristic peak, which is its standard deviation, and c_0 signifies the baseline of the characteristic peak.

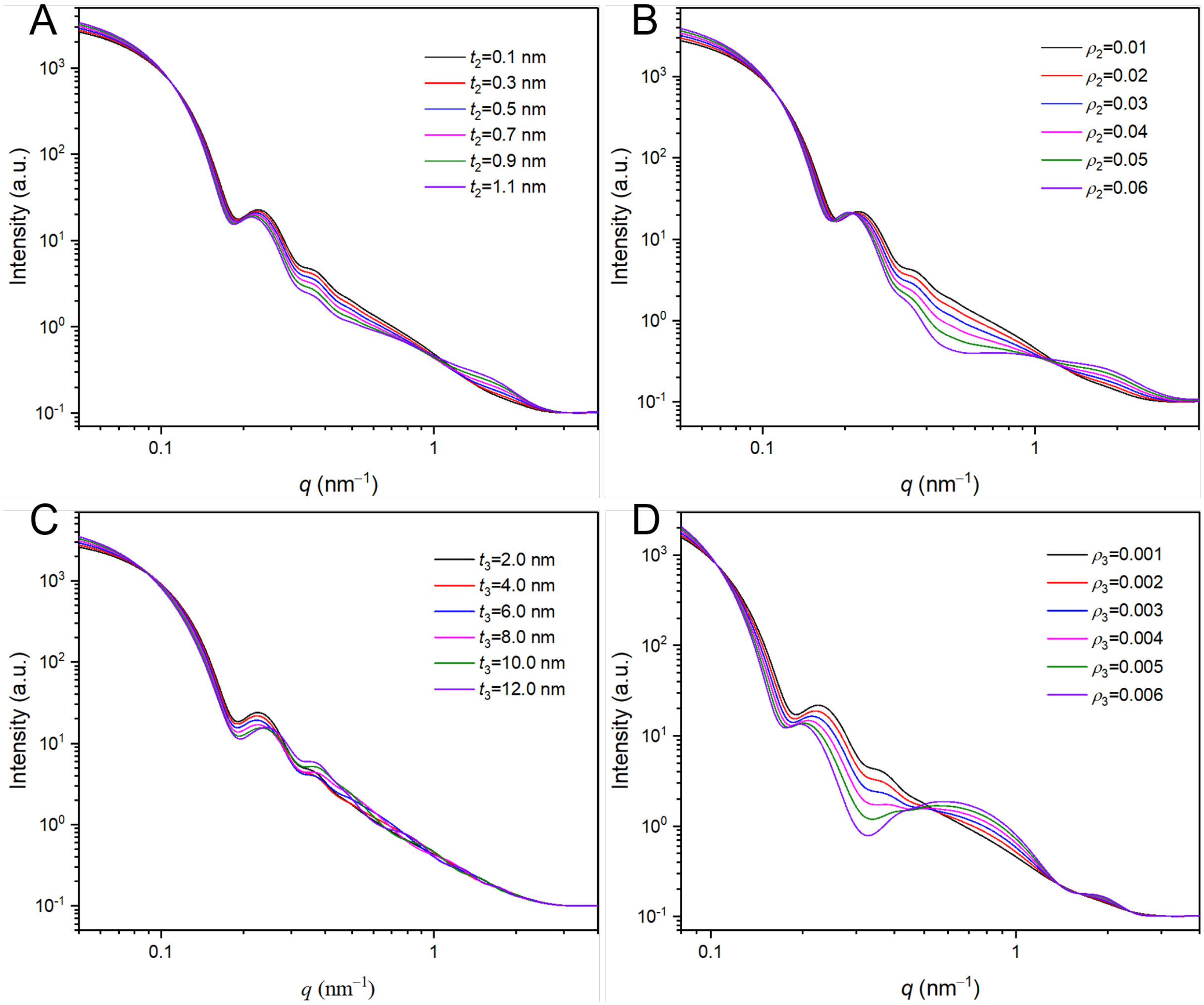

Figure S1. Simulation results of the core-triple shell model where $R_0 = 25$ nm, σ=3 nm, $\rho_c = 0.01$ and $\rho_s = 0$ are fixed (the unit of SLD is arbitrary units): (A) Effect of t_2 on the scattering curves with $\rho_2 = 0.01$; (B) Effect of ρ_2 on the scattering curves with $t_2 = 0.3$ nm; (C) Effect of t_3 on the scattering curves with $\rho_3 = 0.001$; (D) Effect of ρ_3 on the scattering curves with $t_3 = 4$ nm.

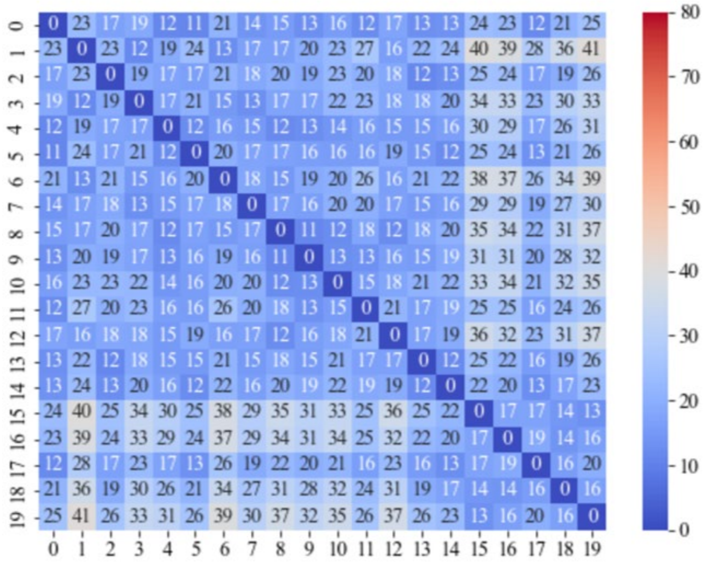

Figure S2. SAXS profile similarity matrix for the evaluated LNPs.
